# The Doctors *v*. the Assurance Societies

**Published:** 1842-04

**Authors:** 


### THE DOCTORS *versus* THE ASSURANCE COMPANIES.

As we find that the question—*Whether medical men are entitled to claim fees when referred to respecting the health of persons seeking to insure their lives?*—is still debated in medical journals and in medical parties, we feel ourselves called on to make a few observations on the subject. Our limits allowing only of the most concise exposition, we shall endeavour to give to our arguments something of the precision of law without its verbosity.

As in this case the Doctors do not seek to establish a claim against their patients, but against the companies applying for information, we shall here consider the Doctor as plaintiff, and the Directors of the Companies as defendants.

On behalf of the former it is urged—

My time is money to me. I never give an opinion without the fee of a guinea, as is well known to those who apply to me. The directors of an office ask me to detail the circumstances of a case which I attended twelve months ago, and request me to state all the circumstances which I consider necessary to enable them to judge of the present state of my patient's health. A compliance with this request must necessarily occupy a considerable portion of my time, for I must have an interview with my patient, to ascertain that I have his authority for disclosing the facts of his case; to refresh my memory as to the circumstances of his case; and to enable me to write an opinion on the present state of his case. After devoting sufficient time for these purposes, I have not only detailed in writing all the circumstances faithfully to the directors, but have expressed my candid opinion as to the best course for them to pursue to effect a profitable contract with the insurer, my late patient; I am, therefore, entitled to my fee, not only in respect of my own loss of time incurred at the special request of the directors, but also on account of the benefit derived by them, from a disclosure of circumstances and the expression of my opinion. *This is the plaintiff's case.*

The defendants contend—

We are not *principals* in this transaction. There is no privity of contract between us and the plaintiff. In the letter addressed to him we stated, that we acted but as *agents* for his patient. The work and labour for which this action is brought, was not done in our retainer. The plaintiff knew this before he incurred the loss of his time; and we have no more to do with this work and labour than with the work and labour in respect of which the plaintiff now requires a bill of parcels through us. If the plaintiff be a physician, he received from the patient a fee for the work at the time it was performed; and if he considers himself entitled, on a *quantum meruit,* to remuneration for a detail of a past transaction the character of which might be properly concealed at the time but which the present interest of the patient requires should be now disclosed; why, let the physician get paid by the patient who has induced us to take the trouble to ask a bill of parcels in his name; a trouble WE incurred out of *delicacy* to the physician, who might prefer giving us a confidential detail of circumstances, to publishing the facts to his patient in person. As far as we are concerned, we neither asked nor desired an examination of the patient by his own physician; to whose opinion, in *this case,* we do not attach the slightest value; as we judge of the proposer's present state from the report of our OWN physician, to whom we pay a fee for advising us, *which fee we do not seek to charge to the proposer.* Then, as to our entering on a profitable contract, the result must determine that fact: it promises to be profitable, or we should not enter into it; and we do all that becomes honest men, meaning to perform their own engagements, when we endeavour to ascertain the trustworthiness of the party before we enter into a contract with him.

Suppose a man want credit, must he not prove his circumstances to be trustworthy before he can obtain it? Suppose he purchase a Government annuity, must he not give evidence of his age before it be granted to him? Would the tradesman remunerate the referee of his customer? or Government pay the cost of the baptismal certificate? Certainly not. Why then should an insurance office bear the expense of proving the health of a proposer to be trustworthy, as a condition precedent to his obtaining a contract for a large sum payable at his

LIBRARY OF THE

Orleans Parish Medical Society death, for a small annuity during his life? If we, the defendants, had adopted the plaintiff as our adviser, and paid him as such, should we not be told from the Bench—"You cannot question the truth of the physician's statements in a court of law. You employed him yourselves—you took the case out of the hands of the proposer, who was bound to make out his health as trustworthy by medical testimony, and you bribe his physician for a report to which you now object? You have made yourselves principals, and the physician *your* agent, instead of his being the agent of the proposer?"

*This is the case on the behalf of the defendants;* and whatever has been urged by them as against a plaintiff who is a physician, they repeat with double force when the claimant is an apothecary.

Now, as impartial judges on these statements, in which there is no issue as to *facts*, but a mere appeal to our legal or moral consideration, we do not hesitate to pronounce FOR THE DEFENDANTS. Indeed, if the question were simply between medical adviser and patient, we should say that it would be beneath the dignity of a liberal profession to exact a fee for the mere particulars of services rendered and paid for. The phrase "Bills of Parcels," which we have heard used in these discussions, is homely, but pertinent; and for such details we think even the customer ought not to pay: but to seek to charge an independent party with a price for this *invoice*, certainly appears to us as little short of absurd.

